# Decoding English Alphabet Letters Using EEG Phase Information

**DOI:** 10.3389/fnins.2018.00062

**Published:** 2018-02-07

**Authors:** YiYan Wang, Pingxiao Wang, Yuguo Yu

**Affiliations:** ^1^State Key Laboratory of Medical Neurobiology, School of Life Science and the Collaborative Innovation Center for Brain Science, Center for Computational Systems Biology, Institutes of Brain Science, Fudan University, Shanghai, China; ^2^Institute of Modern Physics, Fudan University, Shanghai, China

**Keywords:** brain-computer interface, support vector machine (SVM), human brain, theta-band oscillation, visual cortex

## Abstract

Increasing evidence indicates that the phase pattern and power of the low frequency oscillations of brain electroencephalograms (EEG) contain significant information during the human cognition of sensory signals such as auditory and visual stimuli. Here, we investigate whether and how the letters of the alphabet can be directly decoded from EEG phase and power data. In addition, we investigate how different band oscillations contribute to the classification and determine the critical time periods. An English letter recognition task was assigned, and statistical analyses were conducted to decode the EEG signal corresponding to each letter visualized on a computer screen. We applied support vector machine (SVM) with gradient descent method to learn the potential features for classification. It was observed that the EEG phase signals have a higher decoding accuracy than the oscillation power information. Low-frequency theta and alpha oscillations have phase information with higher accuracy than do other bands. The decoding performance was best when the analysis period began from 180 to 380 ms after stimulus presentation, especially in the lateral occipital and posterior temporal scalp regions (PO7 and PO8). These results may provide a new approach for brain-computer interface techniques (BCI) and may deepen our understanding of EEG oscillations in cognition.

## Introduction

The past decade has witnessed great developments in brain–computer interfaces (BCIs), aiming to help severely physically impaired patients interact with the external world through tasks such as typing letters of the English alphabet on a computer for communication. Studies have applied stimulus-evoked brain electroencephalogram (EEG) or electrocorticography (ECoG) signals, especially event-related potentials (ERPs) with P300 responses (Zhang et al., 2013) and steady-state visually evoked potentials (SSVEP) (Won et al., 2014; Nezamfar et al., 2016), to discriminate stimulus characteristics such as letters. There is increasing evidence that the frequency-related phase pattern and power of neural oscillations may code significant sensory information relevant to human perception of the external world, especially in low-frequency bands (Luo and Poeppel, 2007; Schyns et al., 2011; Wang et al., 2012; ten Oever and Sack, 2015). Luo et al. (Luo and Poeppel, 2007) demonstrated that the phase pattern of theta-band (5–8 Hz) activities from the human auditory cortex contains information used to discriminate spoken sentence signals. Their findings indicated a approximately 200 ms time window (approximately 5 Hz within the theta rhythm) that may be critical for discrete perceptive processes. Subsequent phase-decoding studies in audio perception have observed that a similar oscillation frequency range (3~7 Hz) is dominant in spoken sentence recognition (Luo and Poeppel, 2007; Howard and Poeppel, 2010; Wang et al., 2012; Ng et al., 2013; ten Oever and Sack, 2015). Ng et al. (2013) demonstrated that stimuli can be discriminated by the firing rates and phase patterns but not by the oscillation amplitude. Another recent study presented evidence that syllables with varying visual-to-auditory delays are preferably processed at different oscillatory phases (ten Oever and Sack, 2015). Wang et al. (2012) employed the scalp tangential electric field and the surface Laplacian operator around the auditory cortical area to improve the recognition rate of English phonemes. They built a complicated bootstrap-based method that achieved 53% accuracy for all eight phonemes and showed that phase sequences performed better. also revealed that changes in the amplitude (Worden et al., 2000; van Dijk et al., 2008) and phase (Vanrullen et al., 2011) of ongoing alpha activities (9–12 Hz) several hundred milliseconds before a stimulus can modulate the visual discrimination level. In fact, more recent evidence suggests that decreased alpha power may be tightly correlated to the increase in the visual baseline excitability level, which may serve to improve task performance (Lange et al., 2013; Iemi et al., 2017).

The above studies suggest the importance of the frequency, phase, and amplitude of slow oscillatory activities in object representation and categorization (Fries et al., 2007; Schyns et al., 2011). For example, the oscillatory power of various frequency bands may serve to modulate sensory excitability and attention (Klimesch, 1999; Engel et al., 2001; van Dijk et al., 2008), while oscillatory phase patterns across theta and gamma bands may be engaged in information processing, visual attention and working memory (Lisman and Idiart, 1995; Siegel et al., 2009; Heusser et al., 2016).

In this study, we examined the possibility of employing EEG phase and power signals to discriminate input stimulus for a brain-computer interface (BCI) approach. We chose the English alphabet as the visual stimulus because it is a “model” stimulus in BCI research. Based on the above experimental studies (Luo and Poeppel, 2007; van Dijk et al., 2008; Busch et al., 2009; Canolty and Knight, 2010; Schyns et al., 2011; VanRullen and Macdonald, 2012; Wang et al., 2012; ten Oever and Sack, 2015; Watrous et al., 2015; Heusser et al., 2016; Tomassini et al., 2017), which presented evidence on how the oscillatory parameters (phase, power, and frequency) may code visual and auditory information, we hypothesize that information from the visual presentation of different letters in the English alphabet may be encoded in EEG low-frequency phase patterns. Phase decoding and statistical machine-learning analysis may be a novel method, in addition to the traditional ERP method, for discriminating visualized letters. This may be of great benefit for the development of BCI techniques. In addition, it is believed that visual information first flows through the primary visual cortex and then up to higher levels such as V3/4 TEO and TE, which is called the ventral pathway in object recognition tasks (Tanaka, 1996; Krüger et al., 2013). The ventral pathway was thought to be particularly important for reading, including word and letter recognition (Price and Devlin, 2011). Therefore, we questioned whether there was a classification accuracy difference between the scalp occipital and scalp tempo-occipital regions. To examine the above issues, a simple BCI protocol was designed in which subjects watched randomly selected letters on a computer monitor. EEG data were collected from each subject, and an analysis was applied to determine whether visual letter stimuli could be discriminated based on the EEG phase pattern and power amplitude.

## Materials and methods

### Subjects

Fourteen right-handed students from Shanghai Fudan University were recruited by providing monetary compensation. Right-handedness was determined using the Edinburgh handedness inventory (Oldfield, 1971). All subjects (nice males and five females, mean age 25.4, range: 21–32) had normal color vision, corrected visual acuity and no history of neurological or psychiatric problems. This study was approved and supervised by the Ethics Committee of the School of Life Sciences at Fudan University (No. 290). All participants signed written informed consent.

### EEG recordings and experimental design

The EEG data were recorded with a 500 Hz sampling rate in a sound-proof room using a 64-channel actiCHamp Brain Products recording system (Brain Products GmbH, Inc., Munich, Germany) relative to a Cz reference signal. The ground electrode was placed on the Fz electrode. The impedance levels were maintained below 10 kohm.

The stimuli were presented using a pre-programmed e-prime protocol. Five lowercase letters, “a,” “e,” “I,” “o,” and “t” were chosen as the letters to be visually presented on the computer screen. The letter “t” was chosen to exclude pronunciation peculiarity because the remaining four letters were vowels. The letters were in white Times New Roman font and presented on an approximately 12 cm^*^12 cm black background, in a field of view (FOV) of 6.88 degrees. The subjects sat one meter away from a 23-inch screen. The screen was adjusted as high as the height of the seated subject so that the subjects could keep their eyes horizontal. The subjects were directed to focus on the screen and not to move their heads. When a letter was presented, the subjects were directed to read it silently without mouth action. This was intended to keep the subject focused and to avoid any myoelectric artifacts. The participants were instructed to minimize eye movements during the visual presentation and to fixate on the center.

**Figure 1:**
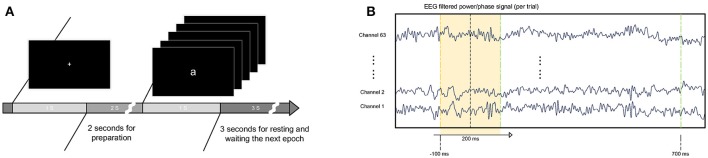
Experimental procedure and data processing. **(A)** Letters were shown randomly in the center of the screen in white on a black background. The letters were approximately 12 cm*12 cm. The letters were presented for 1 s, followed by a 3-s resting period. Before presentation of the letter, a white cross was shown on the screen for subjects to maintain eye fixation. **(B)** Training sets were extracted from filtered EEG power/phase signals with a fixed window length of 200 ms. The window began 100 ms before the appearance of the letter and ended 500 ms later.

Figure 1 presents the experimental protocol. In each trial, a randomly displayed letter appeared on the screen for 1 s and was followed by a 3-s blank interval. Before the appearance of the letter, the subjects were directed to focus their eyes on a white cross on the screen for 1 s. In the study, the subject watched five letters appear individually randomly for 450 trials. The 450 trials were divided into three blocks, with each block containing 150 trials. At the beginning of each block, an instruction was presented on the screen, and the program was paused until the subject pressed the “enter” button to continue. In each block, the letters randomly appeared 150 times, with each letter for 30 times. Between each block, the subject had a short break and then chose when to continue the next study block. It took approximately 60 min to finish three blocks. Between each block, the recording was paused, and the electrode conductance was examined. The mean of the successful trials used for analysis is 351 ± 55 *(*mean and SD) over all subjects.

### Data preprocessing analysis

Data preprocessing analysis was performed using EEGLAB (Delorme and Makeig, 2004) and included bandpass filtering (0.5–220 Hz), epoch extraction locked to the onset of the letters (−500 to 1,000 ms) and baseline correction (−500 to 0 ms). To avoid confusion, we called these data “wide-band data” to differentiate them from the later narrow-band filtered data such as the alpha band EEG data. Signal artifacts were removed in two steps. First, the data were visually inspected, and epochs containing artifacts such as extremely high-amplitude electrode cable movement-induced fluctuations were rejected. Second, epochs containing typical eye movements and eye-blink artifacts that occurred during the first 800 ms after the onset of the letters were rejected. An independent component analysis (ICA) was applied to decompose the EEG data. After decomposition, 63 time-sequence data of component activations were obtained that corresponded to 63 recording channels for each subject. These component activations were recognized as EEG activity or non-brain artifacts by visual inspection of their scalp topographies, time courses, and frequency spectra. The artifact components related to heart beats, temporal muscle movement, eye movements and eye blinks were removed. The criteria for categorizing component activations as EEG activity included the following: (1) spectral peak(s) at typical EEG frequencies and (2) similar responses across each trials; i.e., an EEG response should not occur in a small number of trials only (Delorme and Makeig, 2004). Based on these criteria, the component activations representing non-brain artifacts were removed (the removed ICAs are 11.07 ± 8.62, mean and SD, for 14 subjects), and the EEG data were reconstructed from the remaining component activations.

We then employed the Hilbert transform to convert the real-time artifact-cleaned EEG sequence into a complex time sequence. Each complex number has amplitude and angle information. We derived the amplitude sequence A(t) and phase sequence P(t) separately. Then, we applied machine-learning analysis based on the amplitude or phase sequence data. The formula for the Hilbert transform is presented here:

$$\begin{array}{l} Y\left(t\right)\;=H\left(x\left(t\right)\right)\;=\;\overset{+\infty }{\underset{-\infty }{\int }}x\left(\tau \right)*\frac{1}{t-\tau }d\tau \end{array}$$

Hilbert transformation converts the raw real signal into an imaginary counterpart, and these two parts make a complex signal. The power sequence is defined as the magnitude of this complex signal, and the phase sequence is its phase angle.

Moreover, delta (1–4 Hz), theta (4–8 Hz), alpha (8–14 Hz), beta (14–30 Hz), and gamma (30 Hz above) band oscillations are five typical rhythms observed in the cortex and are thought to be closely related to cognition processes (Kahana et al., 2001; Colgin et al., 2009; Fries, 2015). Additionally, the gamma oscillation can be further divided into low-gamma (30–50 Hz) and high-gamma (50–150 Hz) oscillations. To investigate the functional role of these oscillations in letter classification performance, the original epoched EEG response was filtered into these six bands using a Kaiser window linear phase FIR filter in the MATLAB FDA toolbox. The stop bands were set to attenuate the signal magnitude at −30 dB with a 1 Hz edge band. A Hilbert transformation was then applied to the filtered data.

### Multi-class classification analysis and gradient ascent approach

Five-class classification was employed to discriminate the five letters and to investigate the possibility that the EEG phase pattern or power pattern could be used as a feature in EEG-based BCI. A supervised machine-learning algorithm, LIBSVM, a library for support vector machine (SVM) classifiers (Chang and Lin, 2011), was used and implemented in the MATLAB toolbox. The classifications were quinary with a chance level of 20 percent, and the results of these quinary predictions were evaluated electrode by electrode. The Gaussian function was used as the nonlinear transform function in the SVM classifier, and its critical parameter sigma was determined using a gradient ascent approach, which is similar to the steepest descent algorithm, in which the parameter is adaptively adjusted according to the changes in classification accuracy to ensure that it can be maximized. According to previous research (Schyns et al., 2011), visual stimuli-evoked EEG responses were most informational in the occipital and occipital-temporal cortices. Therefore, the focus was on these 17 electrode sites: P7, P5, P3, P1, Pz, P2, P4, P6, P8, PO7, PO3, POz, PO4, PO8, O1, Oz, and O2. The additional methodological steps encompassing the computational strategy for validating the classification results (cross-validation and shuttered label training sets) are described below.

### Cross-validation approaches and shuffled-label training sets

Cross-validation of the multiclass classification analysis was conducted to obtain robust estimates of the discrimination accuracies and to test the generalization ability of our classifier. In this study, a 30-fold cross-validation approach was adopted. The EEG signal sets were randomly divided into 30 parts, and 29 parts were chosen to train the SVM, which was subsequently used to test the remaining set to obtain the discrimination accuracy (Please note that there are total 450 trials corresponding to five letters for one subject. The 450 trials were divided into 30 parts, with each part contains 15 trials for five letters). This procedure was repeated 30 times, averaging each repetition's accuracy to obtain the final accuracy. To exclude the artificial classification effect caused by the adoption of the SVM classifier and to estimate the validity of the classification result, the labels that indicated the letter for each trial were randomly shuffled 100 times to form 100 random label-training sets. A multiclass classification analysis with a 30-fold cross-validation approach was used on these random label-training sets, and a random label training result ensemble was obtained. In each turn, a subject was randomly selected and the labels of the letters was randomly shuffled. After that, we chose the highest classification accuracy across the electrodes. And then we did this process one-hundred times. Which means we had 100 random-labeled accuracies. We called this a random-label classification accuracies ensemble. A Kolmogorov-Smirnov test (K-S test) was conducted on this ensemble to determine whether the ensemble satisfies a supposed distribution, such as a norm distribution, and if so, to determine its mean value and variance. Finally, the statistical significance was calculated (*p* < 0.0013, three sigma standard) based on the mean and variance of this permuted accuracy.

For comparisons of classification accuracy difference between phase and power groups data of 17 electrodes with 12 subjects, we have performed two-way anova analysis and then performed all the pairwise comparisons using Tukey-Kramer's multiple compare method (Specifically, we first applied [p,~,stats] = anova2(data,12) in Matlab. Data is a 24^*^17 matrix, with the first 12 lines are power accuracy values from 12 subjects, while lines from 13 to 24 are phase accuracy values from 12 subjects; and 17 corresponds to 17 electrodes. Then we have performed multiple comparison with: C = multcompare(stats) in Matlab, default is Turkey-Kramer method). Tukey-Kramer Multiple comparison method is one of the best methods for all-possible pairwise comparisons of group means, to determine which are significantly different from which others. Multiple comparison procedure was performed for significant analysis of pairwise comparison results.

To understand the analysis procedure in a clear way, please see the flowchart Figure S2.

## Results

### Classification accuracy for wide-band EEG phase and power sequences

The power and phase sequences were both 1,500 ms (starting at −500 ms before the appearance of “letter” and stopping at the end of “letter”), and a short 200 ms portion (starting at the 100th ms after the appearance of “letter”) was selected for classification accuracy analysis. The reason starting at the timing of 100th ms is based on the following analysis result.

**Figure 2:**
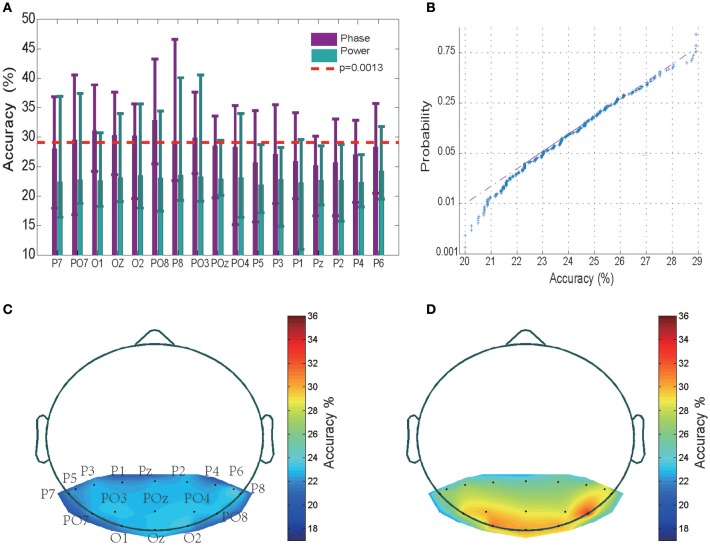
Classification results and accuracy topography. **(A)** Mean classification accuracies across 12 subjects in 17 electrodes. The error bar indicates the upper and lower limit of the accuracy. The performance of the EEG phase and power portions are represented in purple and green, respectively. The red dashed line represents the three-sigma level above the chance level. **(B)** Normplot figure for random label training set classification results. The Y axis indicates the logarithm of the cumulative density function (CDF). If a sample set originates from a normal distribution, it will be linear. **(C)** Accuracy topography for the EEG power portion. The small black dots represent electrodes. Accurate rates at other sites were determined using the MATLAB Triangle interpolation function. **(D)** Accuracy topography for the EEG phase portion.

The timing of the appearance of a “letter” is set as 0th ms. Using this 0th ms timing as the starting point, we chose the sequence of different sizes of time window to examine where the valuable information is started to be encoded. The tested time period is from 0 to 600 ms with time step equal to 2 ms. We observed that the classification accuracy is around chance level for the time period <100 ms, while the accuracy increased rapidly to a 31% high value as the time period was increased to 200 ms, and then fluctuated to reach a saturation level when the time period was further increased to 600 ms (see Figure S1). This analysis suggests that the the EEG sequence <100th ms may not contain valuable information. Therefore, in the following, the classification accuracy values were obtained by training a SVM classifier using 200 ms EEG power/phase sequences that started at the 100th millisecond after presentation of a letter. The mean and variance of the classification accuracy of each of the 17 electrodes for all 12 subjects are shown in Figure 2A (data for the remaining 2 subjects without significant classification power are shown separately in Figure S3). The highest accuracy was 46.61% (chance level of 20%) for a wide-band (0.5–220 Hz) EEG phase sequence (Figure 2A). The EEG phase sequence in 17 electrodes of 12 subjects (28.42 ± 3.21, mean ± SD) showed significantly higher correct rates than the EEG power sequence (22.89 ± 3.02, mean ± SD) at a *p* < 10^−9^ confidence level (two way ANOVA analysis with Tukey-Kramer multiple comparison correction conducted in MATLAB). This implies that the EEG phase portion contains more information than EEG power portion. Multiple comparison procedure was performed for significant analysis of pairwise comparison results, and PO8 was observed to have significantly higher accuracies than P1, P2, P5, Pz (0.01 < P < 0.05) while no significant difference was observed between any pair of accuracy values of other electrodes for phase sequences. The confidence interval was determined using the variance of a fully random shuffled label training set classification accuracy. Figure 2B shows the normplot figure for random label training set classification results. The Y axis indicates the logarithm of the cumulative density function (CDF). The regression linear fitting analysis suggests that the classification accuracy values <29% are mainly from a normal distribution (K-S test *p* = 0.038). The mean was 23.81%, and the variance was 1.76%; thus, the three-sigma level was 29.09%. This value was set as the confidence interval with a one-tail confidence level *P* = 0.0013 (see red dashed line in Figure 2A). We observed that 12 of the 14 subjects with 450-trial tests had significant classification power above the three-sigma level, with 29.09% accuracy in at least one electrode; further, 8 subjects had three electrodes, and seven subjects had five powerful electrodes that showed significant classification power >29.09%. We also conducted phase and power decoding analyses of the data from the 2 subjects who did not have electrode data with significant classification effects (see Figure S3). The highest accuracy for these subjects was only 29% for the phase classification (Figures S3A,B) and 27% for the power classification (Figure S3C). The mean accuracy value of the phase decoding for all 17 electrodes for the 12 subjects was 28.42 ± 3.21 (mean ± SD) and 27.71 ± 3.45 for all 14 subjects. Hence, the following results analysis were mainly based on the 12 subjects. The analysis of the 2 subjects with no significant effects are shown separately in Figures S3, S4.

As is shown in Figures 2C,D for the averaged spectrum of 12 subjects with at least 1 electrode with significant classification power, the relatively high classification accuracy appeared in electrodes placed in the left and right posterior regions.

### Different EEG frequency bands and period-specific classification results

**Figure 3:**
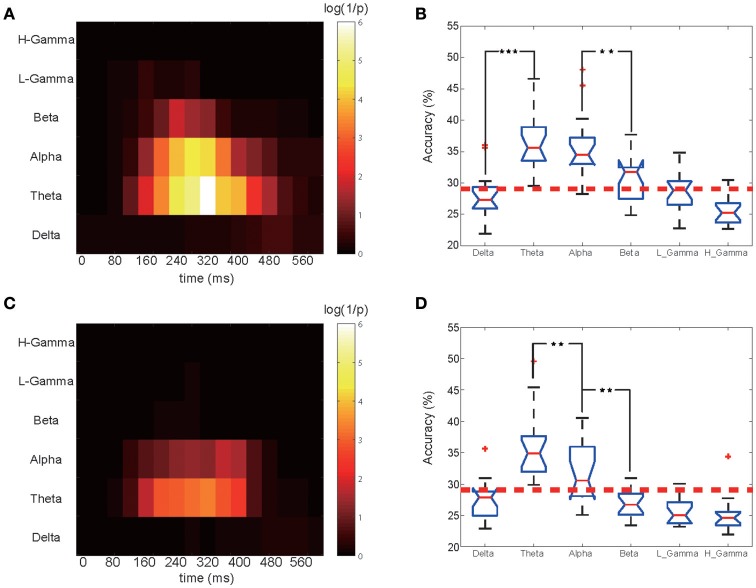
Time-frequency classification significance diagram and bands comparison. **(A)** Classification significance for the EEG phase portion in different bands and time periods. for the 12 subjects shown in Figure 2. Each small block represents a 200 ms training set. For a particular band and time period, the highest accuracy among all 17 electrodes was chosen and its corresponding *P*-value was calculated. The X ticks indicate each periods midpoint, from 0 to 600 ms. **(B)** Comparison of the EEG phase portion classification performance for all bands for the selected optimal time period. One star corresponds to a *P* < 0.05 significance level for the related two bands, and three stars corresponds to *P* < 0.001. **(C)** Classification significance for the EEG power portion in different bands and time periods. **(D)** Comparison of the classification performance of the EEG power portion of all bands.

**Figure 4:**
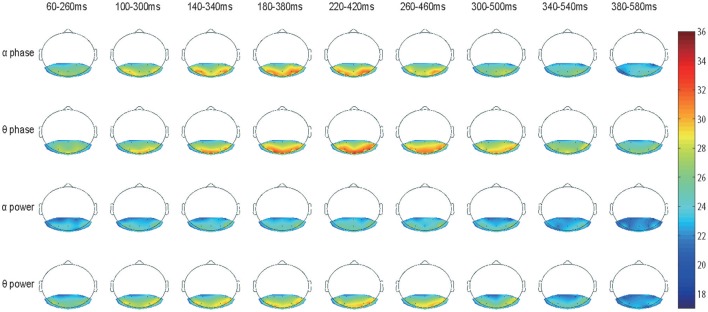
Accuracy topography of time series. Three special sets, the EEG theta power, the theta phase and the alpha phase, were selected for plotting as they had significantly stronger classification power than the others. The EEG theta phase signals clearly had the best performance with long-lasting classification power, the larger useful area, and the highest accuracy rate.

To examine the critical period for classification, a shifting 200 ms-long window (from −100 to 500 ms, 40 ms per step) was applied to the frequency-filtered power and phase time-courses to extract the training and test sets. We observed that the discrimination accuracy within the first 100 ms period after the presentation of a letter is always approximately equal to chance, while most of the valuable decoded information is in the first half-second period (100–600 ms) after the stimuli's presentation (see Figures 3, 4). Hence, our analysis suggested that starting at the 100th millisecond mark after the presentation of a letter may result in a higher classification power than analysis starting from 0 ms after the presentation of a letter (van Gerven et al., 2013; Watrous et al., 2015).

The training and classification processes were employed on these frequency- and time-specific phase signal and power sets to calculate the mean accuracy over 12 subjects in which we obtained significant results in the previous analysis step. A 2-dimensional accuracy matrix was obtained with the X ticks representing the medial time point of each shifting window and the Y ticks representing all six bands. The classification accuracies were transformed into their *P*-value representations. The P value was calculated as the probability that the frequency-filtered power and phase time-courses' accuracy rate can occur from a norm distribution that we obtained from the shuffled label training sets. For a higher accuracy rate, a smaller *P*-value would be obtained. A denary logarithm of 1/P was calculated and chosen as the presentation of the classification performance for illustration purposes. We next compared each frequency band's performance. We selected the best performing time block for each band. Figures 3A,C shows the calculated classification significance as a function of time for six bands. Using the calculation, the best performing time block was chosen based on the highest classification significance level for each frequency band, and the corresponding accuracy value was obtained for the same time block. Then, we applied the MATLAB ANOVA toolbox to examine whether these six bands' signals had significantly different classification performance. The EEG phase signal and power signal portions were treated separately.

The phase and power information in different EEG oscillatory band frequencies that contribute to the classification were also studied. Figure 3A shows the results of the calculation of the classification significance based on the EEG phase signal, and Figure 3B shows a quantification of its classification performance for the 12 subjects who had significant classification power (data for the remaining 2 subjects without significant classification power are shown in Figure S4). The X ticks represent the mid-time point of each shifting 200-ms-long window, which started at 0 ms and ended at 600 ms. As shown in Figure 3A, the higher the logarithm value, the higher the accuracy rate it represents. We also calculated the classification significance and performance values based on the EEG power information (Figure 3C). For both the EEG power and the phase coding performance, the theta frequency band showed higher classification performance than did the remaining five bands, and the crucial time period began at 60 ms to 580 ms (with a middle time point of 160–480 ms). We found that for theta band, phase part and power part had no significant difference (MATLAB ttest2, *P* = 0.89). While in alpha band, phase sequence had a significantly higher accuracy than its power counterpart (ttest2, *P* = 0.0341). Also the beta band performed differently (*P* < 0.001).

For both the theta and the alpha frequency bands, the significance and performance levels are generally relatively lower in the power coding than the phase coding (Figure 3C). The highest accuracy appeared in the period from 220 to 420 ms for phase coding at the theta band and at 180 to 380 ms for the alpha band.

Figures 3B,D shows the calculated classification accuracy for different frequency bands based on EEG oscillatory phase and power components. The EEG rhythmic frequencies significantly influenced the classification accuracy [*F*_(5. 96)_ = 22.64, *P* < 10^−6^ MATLAB ANOVA1]. Figure 3B shows that, for EEG phase coding, there was no significant difference in classification between the theta (36.70 ± 4.43, mean ± SD) and alpha bands (35.4 ± 4.21), but there was a significant difference between the alpha (35.40 ± 4.21) and beta bands (30.74 ± 4.32) (*p* = 0.0037, ANOVA1) for the 12 subjects. Figure 3D shows that, for power coding, the EEG theta band (35.08 ± 5.32) accuracy was significantly higher than the alpha band (31.67 ± 4.29) accuracy and that the alpha band accuracy was significantly higher than that of the other four frequency bands. The remaining four bands did not show a significant classification effect. In addition, if the data analysis includes the two non-significant subjects, the phase decoding accuracy value for the theta band for all 14 subjects was 35.50 ± 5.08, which was slightly lower than the 36.70 ± 4.43 result for the 12 subjects.

### Accuracy topology map for shifting time window data

Based on our current decoding methods, we would like to examine the spatial-temporal distribution of classification accuracy values. Here, we focus on the alpha and theta bands because they showed significantly high classification accuracy (Figures 3B,D). The accuracy values from the 12 subjects were averaged and represented in color (see Figure 4). Figure 4 shows the classification accuracy map derived from both phase and power information in the alpha and theta bands for the 17 electrodes as a function of time.

Unlike the results shown in Figure 2D, there was no strong accuracy lateralization for right hemisphere electrodes, only slightly longer lasting classification power (e.g., the alpha band phase signal from 260 to 460 ms and the theta band phase signal from 300 to 500 ms). The classification power of electrode PO7 had faded but was still in electrode PO8). Interestingly, electrodes O1, O2, and O3 also achieved very high accuracy rates, as PO7 and PO8 did in the theta band phase signal, but presented low values in the alpha band. This difference implies that the theta and the alpha signals may play distinct roles in recognition and have different origins (Fries, 2015).

The classification power in all 17 electrodes clearly faded after 380 ms, and the accuracy decreased to a chance level. Therefore, the remaining topographic maps are not shown.

## Discussion

### Comparison with existing BCI methods and other phase coding research

This study revealed that the phase patterns and power in the theta and alpha bands may contain valuable information about the input stimulus features. This valuable temporal phase coding approach was confirmed with a conclusion consistent with the most recent investigations into decoding other visual and auditory signals in multiple behavior and cognition tasks (Luo and Poeppel, 2007; Schyns et al., 2011; Vanrullen et al., 2011; Wang et al., 2012; ten Oever and Sack, 2015). In addition, decoding of phase and power sequences in different frequency bands suggests different classification powers. Decoding the phase patterns in theta and alpha oscillations provided relatively higher discrimination accuracy than did the delta, beta and gamma band oscillations. Previous studies suggested that the ventral occipital-temporal (vOT) cortex is involved in the perception of visually presented objects and written words (Dahaene, 1995; Price and Devlin, 2011; Matsuo et al., 2015). Our decoding analysis showed a higher classification power for electrodes placed in occipital-temporal regions compared to other regions, although we should keep in mind that EEG electrodes do not necessarily pick up activity directly under the electrodes. These results provide more evidence to support EEG phase coding in visual perception. Spatially distributed electrodes may encode different preferred stimulus features in this process.

The method used here is not as general as the classic existing BCI methods such as SSVEP and P300 (Zhang et al., 2013; Nezamfar et al., 2016). It also relies on the training of an SVM classifier. The traditional BCI approach often conducts the decoding process in real time. In our approach, we first collected a sufficient amount of EEG response data to input stimuli and then performed the training and decoding processes. In future research, we would expect the faster computer speeds and improved algorithms to allow this decoding approach to occur in real time. In addition, compared with existing BCI approaches, our approach is more reliant on subjects. The performance varied greatly between subjects, similar to the ERD/ERS approach. This implies that we may train the subject in future research to improve the classification performance as in some ERD/ERS research.

Although few studies focus on an EEG phase decoding approach and its performance is not sufficient to evoke more attention, the phase decoding method showed a promising prospect for decoding human brain activities using the mass electromagnetic field. As suggested recently (Panzeri et al., 2015, 2016), this new method and other related methods can be used extensively to improve BMIs, and its performance may be further improved by more sophisticated designs.

Our experimental results are consistent with a previous phase decoding investigation related to an emotional face discrimination EEG experiment (Schyns et al., 2011). Almost similar spatially located electrodes in the theta frequency band and a similar critical time window were obtained. This may suggest a similar cortical pathway involved in the visualization process of alphabet letters and human faces. This similarity also appeared in human fMRI recording (Dehaene and Cohen, 2011). However, in contrast to the face recognition process, our experimental results might include an auditory coding effect in addition to the visualization process. Participants were asked to sit quietly without vocalizing the letters, however, they might read the visualized letters with imaginary pronunciation during the alphabet letter visualization task. The imaginary pronunciation sound duration and intensity might be involved in evoking EEG theta oscillations in the temporal cortex (Luo and Poeppel, 2007; Howard and Poeppel, 2010; Wang et al., 2012; Ng et al., 2013; ten Oever and Sack, 2015) and enhancing psychoacoustic sensitivity (Goswami et al., 2011). Additional experiments must be conducted to identify how much decoded information is purely derived from the visualization process and how much is from an imaginary spoken process. Different from the method of Schyns et al. (2011), we trained an SVM to perform the classification. The merit of this approach is that it may have a potential BCI application, although the present method cannot distinguish how and to what extent the characteristics of the stimuli are encoded into the EEG oscillation phase patterns that might be limited by the spatial and temporal resolution of the EEG signals. Because SVM and other machine-learning methods are a type of black box, more detailed analytical methods and experimental designs must be used in future research to examine the potential value and limitations of this approach.

How low frequency oscillatory phases represent information in visual perception remains an open issue. In audio perception, the evidence indicates that theta oscillation is a mimic to the input speech envelope (Giraud and Poeppel, 2012; Gross et al., 2014). In this case, the peak (phase zero) of the oscillation may represent a high amplitude of speech envelope, and the trough (phase π) is related to the quietness.

In addition, recent studies observed that different neuronal oscillations are not intendent and isolated (Canolty et al., 2006). They can interact with each other to modulate oscillation amplitude and phase patterns, resulting in a cross-frequency coupling effect. The cross-frequency coupling may include several interactions, such as phase synchronization, amplitude co-modulation and phase-amplitude coupling (PAC). PAC is believed to reflect neural coding of signals within the local microscale and macroscale networks of the brain (Canolty and Knight, 2010). There is increasing experimental evidence suggesting that PAC may provide more useful information for decoding of object categories (Watrous et al., 2015; Jafakesh et al., 2016), which need to be deeply studied in future once high quality data of EEG or ECoG recording is available.

## Conclusion

Our experimental results provide strong evidences to confirm that the frequency, phase patterns and power information of cortical oscillation parameters contain important information about stimulus features. First, we found that decoding EEG phase patterns brings higher discrimination accuracy values than decoding EEG power portion. Second, frequency range and cortical spatial location are critical in decoding. We observed that phase patterns of the theta and alpha rhythms recorded in the occipital scalp visual and temporal regions contain more rich information that is valuable for decoding different input visual stimuli compared to other regions. EEG power sequences in the theta oscillation showed a significantly higher discrimination rate than did the chance level, although its classification performance was slightly lower than EEG phase pattern. Decoding the EEG phase and power sequence in the much lower frequency delta band or much higher beta and gamma frequency bands does not result in significant discrimination rates. Third, timing is important. Most of the valuable decoded information is within the first half-second period (100–600 ms) after the stimuli's presentation, and this information is hardly captured by the functional magnetic resonance imaging technique (with a time resolution of approximately 1 s).

In sum, our experimental results support that low-frequency cortical oscillations are actively involved in coding sensory information. Directly decoding the phase and power sequences of EEG signals in the theta band may have great potential in brain-computer interface applications for English alphabet letter discrimination. Although the present EEG study showed that electrodes sited in the occipital scalp visual and temporal regions had higher accuracy rates and always reached the peak first, future research with combined EEG and functional MRI experiments may provide better spatial resolution in distinguishing the precise cortical locations in visual stimulus-encoding sites.

## Author contributions

YY, PW, and YW designed the research, YY and YW performed the research, and YW and YY wrote the paper. All authors reviewed the manuscript.

### Conflict of interest statement

The authors declare that the research was conducted in the absence of any commercial or financial relationships that could be construed as a potential conflict of interest.

## References

[B1] Busch N. A. Dubois J. Vanrullen R. (2009). The phase of ongoing EEG oscillations predicts visual perception. J. Neurosci. 29, 7869–7876. 10.1523/JNEUROSCI.0113-09.2009 19535598 PMC6665641

[B2] Canolty R. T. Edwards E. Dalal S. S. Soltani M. Nagarajan S. S. Kirsch H. E.. (2006). High gamma power is phase-locked to theta oscillations in human neocortex. Science 313, 1626–1628. 10.1126/science.1128115 16973878 PMC2628289

[B3] Canolty R. T. Knight R. T. (2010). The functional role of cross-frequency coupling. Trends Cogn. Sci. 14, 506–515. 10.1016/j.tics.2010.09.001 20932795 PMC3359652

[B4] Chang C.-C. Lin C.-J. (2011). LIBSVM: A library for support vector machines. ACM Trans. Intell. Syst. Technol. 2, 1–27. 10.1145/1961189.1961199

[B5] Colgin L. L. Denninger T. Fyhn M. Hafting T. Bonnevie T. Jensen O.. (2009). Frequency of gamma oscillations routes flow of information in the Hippocampus. Nature. 462, 353–357. 10.1038/nature08573 19924214

[B6] Dahaene S. (1995). Electrophysiological evidence for category-specific word processing in the normal human brain. Neuronreport. 6, 2153–2157. 10.1097/00001756-199511000-00014 8595192

[B7] Dehaene S. Cohen L. (2011). The unique role of the visual word form area in reading. Trends Cogn. Sci. (Regul. Ed). 15, 254–262. 10.1016/j.tics.2011.04.003 21592844

[B8] Delorme A. Makeig S. (2004). EEGLAB: an open source toolbox for analysis of single-trial EEG dynamics including independent component analysis. J. Neurosci. Methods 134, 9–21. 10.1016/j.jneumeth.2003.10.009 15102499

[B9] Engel A. K. Fries P. Singer W. (2001). Dynamic predictions: oscillations and synchrony in top-down processing. Nat. Rev. Neurosci. 2, 704–716. 10.1038/35094565 11584308

[B10] Fries P. (2015). Rhythms for cognition: communication through coherence. Neuron 88, 220–235. 10.1016/j.neuron.2015.09.034 26447583 PMC4605134

[B11] Fries P. Nikolić D. Singer W. (2007). The gamma cycle. Trends Neurosci. 30, 309–316. 10.1016/j.tins.2007.05.005 17555828

[B12] Giraud A.-L. Poeppel D. (2012). Cortical oscillations and speech processing: emerging computational principles and operations. Nat Neurosci. 15, 511–517. 10.1038/nn.3063 22426255 PMC4461038

[B13] Goswami U. Wang H.-L. S. Cruz A. Fosker T. Mead N. Huss M. (2011). Language-universal sensory deficits in developmental dyslexia: English, Spanish, and Chinese. J. Cogn. Neurosci. 23, 325–337. 10.1162/jocn.2010.21453 20146613

[B14] Gross J. Hoogenboom N. Thut G. Schyns P. Panzeri S. Belin P.. (2014). Speech rhythms and multiplexed oscillatory sensory coding in the human brain. PLoS Biol. 11:e1001752. 10.1371/journal.pbio.1001752 24391472 PMC3876971

[B15] Heusser A. C. Poeppel D. Ezzyat Y. Davachi L. (2016). Episodic sequence memory is supported by a theta-gamma phase code. Nat. Neurosci. 19, 1374–1380. 10.1038/nn.4374 27571010 PMC5039104

[B16] Howard M. F. Poeppel D. (2010). Discrimination of speech stimuli based on neuronal response phase patterns depends on acoustics but not comprehension. J. Neurophysiol. 104, 2500–2511. 10.1152/jn.00251.2010 20484530 PMC2997028

[B17] Iemi L. Chaumon M. Crouzet S. M. Busch N. A. (2017). Spontaneous neural oscillations bias perception by modulating baseline excitability. J. Neurosci. 37, 807–819. 10.1523/JNEUROSCI.1432-16.2016 28123017 PMC6597018

[B18] Jafakesh S. Jahromy F. Z. Daliri M. R. (2016). Decoding of object categories from brain signals using cross frequency coupling methods. Biomed. Signal Process. Control 27, 60–67. 10.1016/j.bspc.2016.01.013

[B19] Kahana M. J. Seelig D. Madsen J. R. (2001). Theta returns. Curr. Opin. Neurobiol. 11, 739–744. 10.1016/S0959-4388(01)00278-1 11741027

[B20] Klimesch W. (1999). EEG alpha and theta oscillations reflect cognitive and memory performance: a review and analysis. Brain Res. Brain Res. Rev. 29, 169–195. 10.1016/S0165-0173(98)00056-3 10209231

[B21] Krüger N. Janssen P. Kalkan S. Lappe M. Leonardis A. Piater J.. (2013). Deep hierarchies in the primate visual cortex: what can we learn for computer vision? IEEE Trans. Pattern Anal. Mach. Intell. 35, 1847–1871. 10.1109/TPAMI.2012.272 23787340

[B22] Lange J. Oostenveld R. Fries P. (2013). Reduced occipital alpha power indexes enhanced excitability rather than improved visual perception. J. Neurosci. 33, 3212–3220. 10.1523/JNEUROSCI.3755-12.2013 23407974 PMC6619207

[B23] Lisman J. E. Idiart M. A. (1995). Storage of 7 +/- 2 short-term memories in oscillatory subcycles. Science 267, 1512–1515. 10.1126/science.7878473 7878473

[B24] Luo H. Poeppel D. (2007). Phase patterns of neuronal responses reliably discriminate speech in human auditory cortex. Neuron 54, 1001–1010. 10.1016/j.neuron.2007.06.004 17582338 PMC2703451

[B25] Matsuo T. Kawasaki K. Kawai K. Majima K. Masuda H. Murakami H.. (2015). Alternating zones selective to faces and written words in the human ventral occipitotemporal cortex. Cerebral Cortex 25, 1265–1277. 10.1093/cercor/bht319 24285843

[B26] Nezamfar H. Salehi S. S. M. Moghadamfalahi M. Erdogmus D. (2016). FlashType™: a context-aware c-VEP-based BCI typing interface using EEG signals. IEEE J. Sel. Top. Signal Process. 10, 932–941. 10.1109/JSTSP.2016.2552140

[B27] Ng B. S. Logothetis N. K. Kayser C. (2013). EEG Phase Patterns Reflect the Selectivity of Neural Firing. Cerebral Cortex 23, 389–398. 10.1093/cercor/bhs031 22345353

[B28] Oldfield R. C. (1971). The assessment and analysis of handedness: the Edinburgh inventory. Neuropsychologia 9, 97–113. 10.1016/0028-3932(71)90067-4 5146491

[B29] Panzeri S. Macke J. H. Gross J. Kayser C. (2015). Neural population coding: combining insights from microscopic and mass signals. Trends Cogn. Sci. 19, 162–172. 10.1016/j.tics.2015.01.002 25670005 PMC4379382

[B30] Panzeri S. Safaai H. De Feo V. Vato A. (2016). Implications of the dependence of neuronal activity on neural network states for the design of brain-machine interfaces. Front. Neurosci. 10:165. 10.3389/fnins.2016.00165 27147955 PMC4837323

[B31] Price C. J. Devlin J. T. (2011). The Interactive Account of ventral occipitotemporal contributions to reading. Trends Cogn. Sci. 15, 246–253. 10.1016/j.tics.2011.04.001 21549634 PMC3223525

[B32] Schyns P. G. Thut G. Gross J. (2011). Cracking the code of oscillatory activity. PLoS Biol. 9:e1001064. 10.1371/journal.pbio.1001064 21610856 PMC3096604

[B33] Siegel M. Warden M. R. Miller E. K. (2009). Phase-dependent neuronal coding of objects in short-term memory. Proc. Natl. Acad. Sci. U.S.A. 106, 21341–21346. 10.1073/pnas.0908193106 19926847 PMC2779828

[B34] Tanaka K. (1996). Inferotemporal cortex and object vision. Annu. Rev. Neurosci. 19, 109–139. 10.1146/annurev.ne.19.030196.000545 8833438

[B35] ten Oever S. Sack A. T. (2015). Oscillatory phase shapes syllable perception. Proc. Natl. Acad. Sci. U.S.A. 112, 15833–15837. 10.1073/pnas.1517519112 26668393 PMC4702974

[B36] Tomassini A. Ambrogioni L. Medendorp W. P. Maris E. (2017). Theta oscillations locked to intended actions rhythmically modulate perception. eLife 6:e25618. 10.7554/eLife.25618 28686161 PMC5553936

[B37] van Dijk H. Schoffelen J. M. Oostenveld R. Jensen O. (2008). Prestimulus oscillatory activity in the alpha band predicts visual discrimination ability. J. Neurosci. 28, 1816–1823. 10.1523/JNEUROSCI.1853-07.2008 18287498 PMC6671447

[B38] van Gerven M. A. Maris E. Sperling M. Sharan A. Litt B. Anderson C.. (2013). Decoding the memorization of individual stimuli with direct human brain recordings. Neuroimage 70, 223–232. 10.1016/j.neuroimage.2012.12.059 23298746 PMC3580011

[B39] Vanrullen R. Busch N. A. Drewes J. Dubois J. (2011). Ongoing EEG phase as a Trial-by-Trial predictor of perceptual and attentional variability. Front. Psychol. 2:60. 10.3389/fpsyg.2011.00060 21716580 PMC3110813

[B40] VanRullen R. Macdonald J. S. P. (2012). Perceptual echoes at 10 hz in the human brain. Curr. Biol. 22, 995–999. 10.1016/j.cub.2012.03.050 22560609

[B41] Wang R. Perreau-Guimaraes M. Carvalhaes C. Suppes P. (2012). Using phase to recognize English phonemes and their distinctive features in the brain. Proc. Natl. Acad. Sci. U.S.A. 109, 20685–20690. 10.1073/pnas.1217500109 23185010 PMC3528497

[B42] Watrous A. J. Deuker L. Fell J. Axmacher N. (2015). Phase-amplitude coupling supports phase coding in human ECoG. eLife 4:e07886 10.7554/eLife.07886 PMC4579288 26308582

[B43] Won D.-O. Zhang H. H. Guan C. Lee S.-W. (2014). A BCI speller based on SSVEP using high frequency stimuli design, in 2014 IEEE International Conference on Systems, Man, and Cybernetics (SMC) (San Diego, CA: IEEE), 1068–1071. 10.1109/SMC.2014.6974055

[B44] Worden M. S. Foxe J. J. Wang N. Simpson G. V. (2000). Anticipatory biasing of visuospatial attention indexed by retinotopically specific α-bank electroencephalography increases over occipital cortex. J. Neurosci. 20:RC63. 10704517 10.1523/JNEUROSCI.20-06-j0002.2000 PMC6772495

[B45] Zhang D. Song H. Xu R. Zhou W. Ling Z. Hong B. (2013). Toward a minimally invasive brain–computer interface using a single subdural channel: a visual speller study. Neuroimage 71, 30–41. 10.1016/j.neuroimage.2012.12.069 23313779

