# Supplementary material for: Decoding English Alphabet Letters Using EEG Phase Information
Source: Front Neurosci. 2018 Feb 7;12:62. doi: 10.3389/fnins.2018.00062 (PMC5808334; doi:10.3389/fnins.2018.00062)
Supplement: Supplementary file 1 [file Image1.pdf]

Supplemental information for "Decoding English Alphabet Letters Using EEG Phase Information"

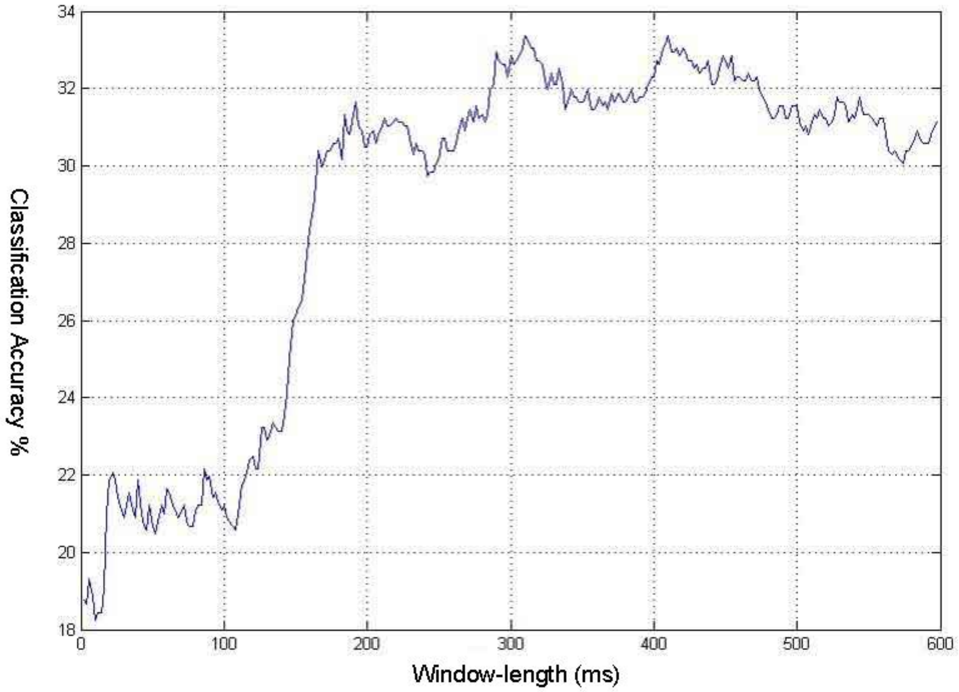

Figure S1.

The effect of selected time window size on the discrimination accuracy for one subject with a fixing starting point. The starting point of the time window was fixed at 0ms and the window size was gradually prolonged with the step 2ms. Noted that at the first 100 ms, the discrimination accuracy was around chance level, while the accuracy increased rapidly to a 31% high value as temporal window was increased to 200 ms, and then fluctuated to reach a saturation level when temporal window size further increased.

Figure S2 flowchart for the data analysis

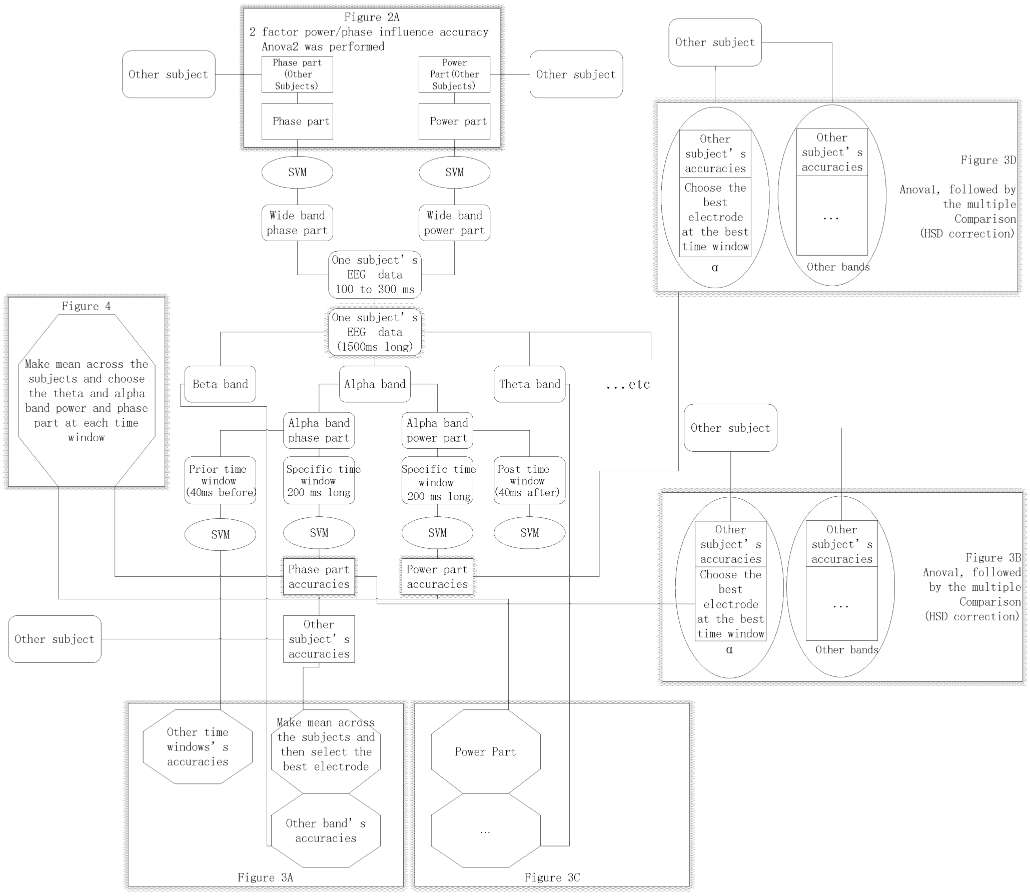

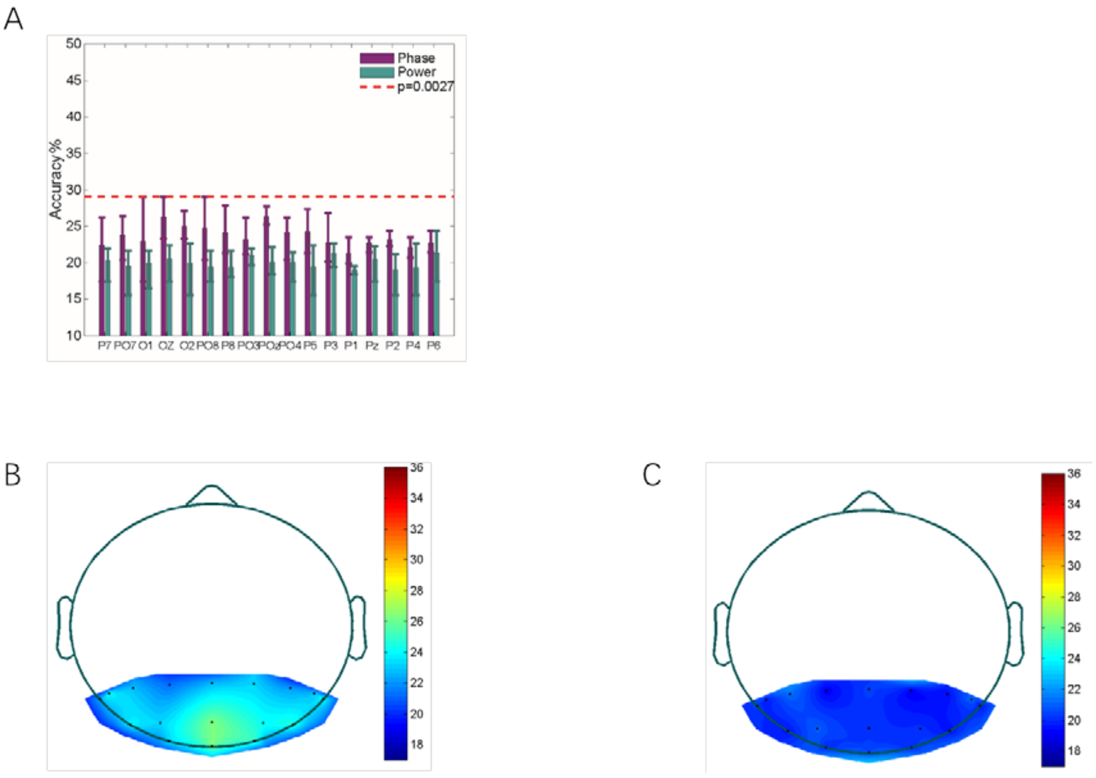

Figure S3. Classification Results and Accuracy Topography for the 2 subjects whose classification accuracy did not reach significant level.

(A) Mean classification accuracies across 2 subjects in seventeen electrodes. The error bar indicates the upper and lower limit of the accuracy. The performance of the EEG phase and power portions are represented in purple and green, respectively. The red dashed line represents the three-sigma level above the chance level.

(B) Accuracy topography for the EEG power portion. The small black dots represent electrodes. Accurate rates at other sites were determined using the MATLAB Triangle interpolation function.

(C) Accuracy topography for the EEG phase part.

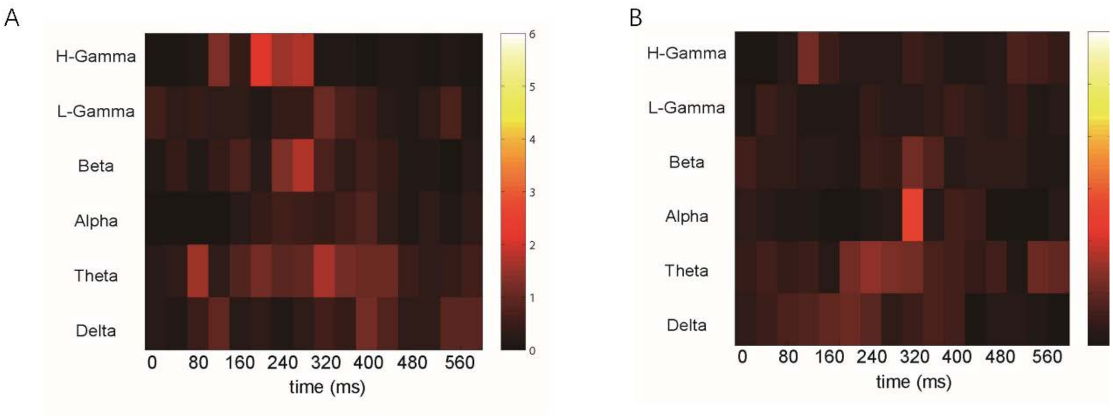

Figure S4 Time-frequency Classification Significance Diagram and Bands Comparison for the 2 subjects whose classification accuracy did not reach significant level.

(A) Classification significance for the EEG phase portion in different bands and time periods for the 2 subjects shown in Figure S2. Each small block represents a 200 ms training set. For a particular band and time period, the highest accuracy among all seventeen electrodes was chosen and its corresponding P-value was calculated. The X ticks indicate each periods' midpoint, from 0 ms to 600 ms.

(B) .Classification significance for EEG power part in different bands and time periods for the 2 subjects same as Figure S2.
